# Supplementary material for: Resolving the full spectrum of human genome variation using Linked-Reads
Source: Genome Res. 2019 Apr;29(4):635–45. doi: 10.1101/gr.234443.118 (PMC6442396; doi:10.1101/gr.234443.118)
Supplement: Supplemental Material [file supp_gr.234443.118_Supplemental_longranger-2.2.zip › longranger-2.2.tar.bz2!/lr2.2-public/lib/stan/cmdstan/stan/doc/api/html/student__t_8hpp_source.html]

Stan: src/stan/prob/distributions/univariate/continuous/student\_t.hpp Source File


|  |  |
| --- | --- |
|  | Stan  2.5.0  probability, sampling & optimization |


- Main Page
- Related Pages
- Namespaces
- Classes
- Files

- File List
- File Members

All Classes Namespaces Files Functions Variables Typedefs Enumerations Enumerator Friends Macros Pages

- src
- stan
- prob
- distributions
- univariate
- continuous

student\_t.hpp

Go to the documentation of this file.

1 #ifndef STAN\_\_PROB\_\_DISTRIBUTIONS\_\_UNIVARIATE\_\_CONTINUOUS\_\_STUDENT\_T\_HPP

2 #define STAN\_\_PROB\_\_DISTRIBUTIONS\_\_UNIVARIATE\_\_CONTINUOUS\_\_STUDENT\_T\_HPP

3

4 #include <boost/random/student\_t\_distribution.hpp>

5 #include <boost/random/variate\_generator.hpp>

6

7 #include <stan/agrad/partials\_vari.hpp>

8 #include <stan/math/error\_handling.hpp>

9 #include <stan/math/constants.hpp>

10 #include <stan/math/functions/square.hpp>

11 #include <stan/math/functions/value\_of.hpp>

12 #include <stan/meta/traits.hpp>

13 #include <stan/prob/constants.hpp>

14 #include <stan/prob/traits.hpp>

15 #include <stan/prob/internal\_math.hpp>

16

17 namespace stan {

18

19  namespace prob {

20

46  template <bool propto, typename T\_y, typename T\_dof,

47  typename T\_loc, typename T\_scale>

48  typename return\_type<T\_y,T\_dof,T\_loc,T\_scale>::type

49  student\_t\_log(const T\_y& y, const T\_dof& nu, const T\_loc& mu,

50  const T\_scale& sigma) {

51  static const char\* function = "stan::prob::student\_t\_log(%1%)";

52

53  using stan::math::check\_positive\_finite;

54  using stan::math::check\_finite;

55  using stan::math::check\_not\_nan;

56  using stan::math::check\_consistent\_sizes;

57

58  // check if any vectors are zero length

59  if (!(stan::length(y)

60  && stan::length(nu)

61  && stan::length(mu)

62  && stan::length(sigma)))

63  return 0.0;

64

65  double logp(0.0);

66

67  // validate args (here done over var, which should be OK)

68  check\_not\_nan(function, y, "Random variable", &logp);

69  check\_positive\_finite(function, nu, "Degrees of freedom parameter",

70  &logp);

71  check\_finite(function, mu, "Location parameter", &logp);

72  check\_positive\_finite(function, sigma, "Scale parameter", &logp);

73  check\_consistent\_sizes(function,

74  y,nu,mu,sigma,

75  "Random variable",

76  "Degrees of freedom parameter",

77  "Location parameter","Scale parameter",

78  &logp);

79

80  // check if no variables are involved and prop-to

81  if (!include\_summand<propto,T\_y,T\_dof,T\_loc,T\_scale>::value)

82  return 0.0;

83

84  VectorView<const T\_y> y\_vec(y);

85  VectorView<const T\_dof> nu\_vec(nu);

86  VectorView<const T\_loc> mu\_vec(mu);

87  VectorView<const T\_scale> sigma\_vec(sigma);

88  size\_t N = max\_size(y, nu, mu, sigma);

89

90  using std::log;

91  using boost::math::digamma;

92  using boost::math::lgamma;

93  using stan::math::square;

94  using stan::math::value\_of;

95

96  DoubleVectorView<include\_summand<propto,T\_y,T\_dof,T\_loc,T\_scale>::value,

97  is\_vector<T\_dof>::value> half\_nu(length(nu));

98  for (size\_t i = 0; i < length(nu); i++)

99  if (include\_summand<propto,T\_y,T\_dof,T\_loc,T\_scale>::value)

100  half\_nu[i] = 0.5 \* value\_of(nu\_vec[i]);

101  DoubleVectorView<include\_summand<propto,T\_dof>::value,

102  is\_vector<T\_dof>::value> lgamma\_half\_nu(length(nu));

103  DoubleVectorView<include\_summand<propto,T\_dof>::value,

104  is\_vector<T\_dof>::value> lgamma\_half\_nu\_plus\_half(length(nu));

105  if (include\_summand<propto,T\_dof>::value)

106  for (size\_t i = 0; i < length(nu); i++) {

107  lgamma\_half\_nu[i] = lgamma(half\_nu[i]);

108  lgamma\_half\_nu\_plus\_half[i] = lgamma(half\_nu[i] + 0.5);

109  }

110  DoubleVectorView<!is\_constant\_struct<T\_dof>::value,

111  is\_vector<T\_dof>::value> digamma\_half\_nu(length(nu));

112  DoubleVectorView<!is\_constant\_struct<T\_dof>::value,

113  is\_vector<T\_dof>::value> digamma\_half\_nu\_plus\_half(length(nu));

114  if (!is\_constant\_struct<T\_dof>::value)

115  for (size\_t i = 0; i < length(nu); i++) {

116  digamma\_half\_nu[i] = digamma(half\_nu[i]);

117  digamma\_half\_nu\_plus\_half[i] = digamma(half\_nu[i] + 0.5);

118  }

119

120

121

122  DoubleVectorView<include\_summand<propto,T\_dof>::value,

123  is\_vector<T\_dof>::value> log\_nu(length(nu));

124  for (size\_t i = 0; i < length(nu); i++)

125  if (include\_summand<propto,T\_dof>::value)

126  log\_nu[i] = log(value\_of(nu\_vec[i]));

127  DoubleVectorView<include\_summand<propto,T\_scale>::value,

128  is\_vector<T\_scale>::value> log\_sigma(length(sigma));

129  for (size\_t i = 0; i < length(sigma); i++)

130  if (include\_summand<propto,T\_scale>::value)

131  log\_sigma[i] = log(value\_of(sigma\_vec[i]));

132

133  DoubleVectorView<include\_summand<propto,T\_y,T\_dof,T\_loc,T\_scale>::value,

134  is\_vector<T\_y>::value

135  || is\_vector<T\_dof>::value

136  || is\_vector<T\_loc>::value

137  || is\_vector<T\_scale>::value>

138  square\_y\_minus\_mu\_over\_sigma\_\_over\_nu(N);

139

140  DoubleVectorView<include\_summand<propto,T\_y,T\_dof,T\_loc,T\_scale>::value,

141  is\_vector<T\_y>::value

142  || is\_vector<T\_dof>::value

143  || is\_vector<T\_loc>::value

144  || is\_vector<T\_scale>::value>

145  log1p\_exp(N);

146

147  for (size\_t i = 0; i < N; i++)

148  if (include\_summand<propto,T\_y,T\_dof,T\_loc,T\_scale>::value) {

149  const double y\_dbl = value\_of(y\_vec[i]);

150  const double mu\_dbl = value\_of(mu\_vec[i]);

151  const double sigma\_dbl = value\_of(sigma\_vec[i]);

152  const double nu\_dbl = value\_of(nu\_vec[i]);

153  square\_y\_minus\_mu\_over\_sigma\_\_over\_nu[i]

154  = square((y\_dbl - mu\_dbl) / sigma\_dbl) / nu\_dbl;

155  log1p\_exp[i] = log1p(square\_y\_minus\_mu\_over\_sigma\_\_over\_nu[i]);

156  }

157

158  agrad::OperandsAndPartials<T\_y,T\_dof,T\_loc,T\_scale>

159  operands\_and\_partials(y,nu,mu,sigma);

160  for (size\_t n = 0; n < N; n++) {

161  const double y\_dbl = value\_of(y\_vec[n]);

162  const double mu\_dbl = value\_of(mu\_vec[n]);

163  const double sigma\_dbl = value\_of(sigma\_vec[n]);

164  const double nu\_dbl = value\_of(nu\_vec[n]);

165  if (include\_summand<propto>::value)

166  logp += NEG\_LOG\_SQRT\_PI;

167  if (include\_summand<propto,T\_dof>::value)

168  logp += lgamma\_half\_nu\_plus\_half[n] - lgamma\_half\_nu[n]

169  - 0.5 \* log\_nu[n];

170  if (include\_summand<propto,T\_scale>::value)

171  logp -= log\_sigma[n];

172  if (include\_summand<propto,T\_y,T\_dof,T\_loc,T\_scale>::value)

173  logp -= (half\_nu[n] + 0.5)

174  \* log1p\_exp[n];

175

176  if (!is\_constant\_struct<T\_y>::value) {

177  operands\_and\_partials.d\_x1[n]

178  += -(half\_nu[n]+0.5)

179  \* 1.0 / (1.0 + square\_y\_minus\_mu\_over\_sigma\_\_over\_nu[n])

180  \* (2.0 \* (y\_dbl - mu\_dbl) / square(sigma\_dbl) / nu\_dbl);

181  }

182  if (!is\_constant\_struct<T\_dof>::value) {

183  const double inv\_nu = 1.0 / nu\_dbl;

184  operands\_and\_partials.d\_x2[n]

185  += 0.5\*digamma\_half\_nu\_plus\_half[n] - 0.5\*digamma\_half\_nu[n]

186  - 0.5 \* inv\_nu

187  - 0.5\*log1p\_exp[n]

188  + (half\_nu[n] + 0.5)

189  \* (1.0/(1.0 + square\_y\_minus\_mu\_over\_sigma\_\_over\_nu[n])

190  \* square\_y\_minus\_mu\_over\_sigma\_\_over\_nu[n] \* inv\_nu);

191  }

192  if (!is\_constant\_struct<T\_loc>::value) {

193  operands\_and\_partials.d\_x3[n]

194  -= (half\_nu[n] + 0.5)

195  / (1.0 + square\_y\_minus\_mu\_over\_sigma\_\_over\_nu[n])

196  \* (2.0 \* (mu\_dbl - y\_dbl) / (sigma\_dbl\*sigma\_dbl\*nu\_dbl));

197  }

198  if (!is\_constant\_struct<T\_scale>::value) {

199  const double inv\_sigma = 1.0 / sigma\_dbl;

200  operands\_and\_partials.d\_x4[n]

201  += -inv\_sigma

202  + (nu\_dbl + 1.0) / (1.0 + square\_y\_minus\_mu\_over\_sigma\_\_over\_nu[n])

203  \* (square\_y\_minus\_mu\_over\_sigma\_\_over\_nu[n] \* inv\_sigma);

204  }

205  }

206  return operands\_and\_partials.to\_var(logp);

207  }

208

209  template <typename T\_y, typename T\_dof, typename T\_loc, typename T\_scale>

210  inline

211  typename return\_type<T\_y,T\_dof,T\_loc,T\_scale>::type

212  student\_t\_log(const T\_y& y, const T\_dof& nu, const T\_loc& mu,

213  const T\_scale& sigma) {

214  return student\_t\_log<false>(y,nu,mu,sigma);

215  }

216

217  template <typename T\_y, typename T\_dof, typename T\_loc, typename T\_scale>

218  typename return\_type<T\_y, T\_dof, T\_loc, T\_scale>::type

219  student\_t\_cdf(const T\_y& y, const T\_dof& nu, const T\_loc& mu,

220  const T\_scale& sigma) {

221

222  // Size checks

223  if (!(stan::length(y) && stan::length(nu) && stan::length(mu)

224  && stan::length(sigma)))

225  return 1.0;

226

227  static const char\* function = "stan::prob::student\_t\_cdf(%1%)";

228

229  using stan::math::check\_positive\_finite;

230  using stan::math::check\_finite;

231  using stan::math::check\_not\_nan;

232  using stan::math::check\_consistent\_sizes;

233  using stan::math::value\_of;

234

235  double P(1.0);

236

237  check\_not\_nan(function, y, "Random variable", &P);

238  check\_positive\_finite(function, nu, "Degrees of freedom parameter", &P);

239  check\_finite(function, mu, "Location parameter", &P);

240  check\_positive\_finite(function, sigma, "Scale parameter", &P);

241

242  // Wrap arguments in vectors

243  VectorView<const T\_y> y\_vec(y);

244  VectorView<const T\_dof> nu\_vec(nu);

245  VectorView<const T\_loc> mu\_vec(mu);

246  VectorView<const T\_scale> sigma\_vec(sigma);

247  size\_t N = max\_size(y, nu, mu, sigma);

248

249  agrad::OperandsAndPartials<T\_y, T\_dof, T\_loc, T\_scale>

250  operands\_and\_partials(y, nu, mu, sigma);

251

252  // Explicit return for extreme values

253  // The gradients are technically ill-defined, but treated as zero

254  for (size\_t i = 0; i < stan::length(y); i++) {

255  if (value\_of(y\_vec[i]) == -std::numeric\_limits<double>::infinity())

256  return operands\_and\_partials.to\_var(0.0);

257  }

258

259  using boost::math::ibeta;

260  using boost::math::ibeta\_derivative;

261  using boost::math::digamma;

262  using boost::math::beta;

263

264  // Cache a few expensive function calls if nu is a parameter

265  double digammaHalf = 0;

266

267  DoubleVectorView<!is\_constant\_struct<T\_dof>::value,is\_vector<T\_dof>::value>

268  digamma\_vec(stan::length(nu));

269  DoubleVectorView<!is\_constant\_struct<T\_dof>::value,is\_vector<T\_dof>::value>

270  digammaNu\_vec(stan::length(nu));

271  DoubleVectorView<!is\_constant\_struct<T\_dof>::value,is\_vector<T\_dof>::value>

272  digammaNuPlusHalf\_vec(stan::length(nu));

273  DoubleVectorView<!is\_constant\_struct<T\_dof>::value,is\_vector<T\_dof>::value>

274  betaNuHalf\_vec(stan::length(nu));

275

276  if (!is\_constant\_struct<T\_dof>::value) {

277  digammaHalf = digamma(0.5);

278

279  for (size\_t i = 0; i < stan::length(nu); i++) {

280  const double nu\_dbl = value\_of(nu\_vec[i]);

281

282  digammaNu\_vec[i] = digamma(0.5 \* nu\_dbl);

283  digammaNuPlusHalf\_vec[i] = digamma(0.5 + 0.5 \* nu\_dbl);

284  betaNuHalf\_vec[i] = beta(0.5, 0.5 \* nu\_dbl);

285  }

286  }

287

288  // Compute vectorized CDF and gradient

289  for (size\_t n = 0; n < N; n++) {

290

291  // Explicit results for extreme values

292  // The gradients are technically ill-defined, but treated as zero

293  if (value\_of(y\_vec[n]) == std::numeric\_limits<double>::infinity()) {

294  continue;

295  }

296

297  const double sigma\_inv = 1.0 / value\_of(sigma\_vec[n]);

298  const double t = (value\_of(y\_vec[n]) - value\_of(mu\_vec[n])) \* sigma\_inv;

299  const double nu\_dbl = value\_of(nu\_vec[n]);

300  const double q = nu\_dbl / (t \* t);

301  const double r = 1.0 / (1.0 + q);

302  const double J = 2 \* r \* r \* q / t;

303  double zJacobian = t > 0 ? - 0.5 : 0.5;

304

305  if(q < 2)

306  {

307

308  double z = ibeta(0.5 \* nu\_dbl, 0.5, 1.0 - r);

309  const double Pn = t > 0 ? 1.0 - 0.5 \* z : 0.5 \* z;

310  const double d\_ibeta = ibeta\_derivative(0.5 \* nu\_dbl, 0.5, 1.0 - r);

311

312  P \*= Pn;

313

314  if (!is\_constant\_struct<T\_y>::value)

315  operands\_and\_partials.d\_x1[n]

316  += - zJacobian \* d\_ibeta \* J \* sigma\_inv / Pn;

317  if (!is\_constant\_struct<T\_dof>::value) {

318

319  double g1 = 0;

320  double g2 = 0;

321

322  stan::math::gradRegIncBeta(g1, g2, 0.5 \* nu\_dbl, 0.5, 1.0 - r,

323  digammaNu\_vec[n], digammaHalf,

324  digammaNuPlusHalf\_vec[n],

325  betaNuHalf\_vec[n]);

326

327  operands\_and\_partials.d\_x2[n]

328  += zJacobian \* ( d\_ibeta \* (r / t) \* (r / t) + 0.5 \* g1 ) / Pn;

329  }

330

331  if (!is\_constant\_struct<T\_loc>::value)

332  operands\_and\_partials.d\_x3[n]

333  += zJacobian \* d\_ibeta \* J \* sigma\_inv / Pn;

334  if (!is\_constant\_struct<T\_scale>::value)

335  operands\_and\_partials.d\_x4[n]

336  += zJacobian \* d\_ibeta \* J \* sigma\_inv \* t / Pn;

337

338  }

339  else {

340

341  double z = 1 - ibeta(0.5, 0.5 \* nu\_dbl, r);

342  zJacobian \*= -1;

343

344  const double Pn = t > 0 ? 1.0 - 0.5 \* z : 0.5 \* z;

345

346  double d\_ibeta = ibeta\_derivative(0.5, 0.5 \* nu\_dbl, r);

347

348  P \*= Pn;

349

350  if (!is\_constant\_struct<T\_y>::value)

351  operands\_and\_partials.d\_x1[n]

352  += zJacobian \* d\_ibeta \* J \* sigma\_inv / Pn;

353  if (!is\_constant\_struct<T\_dof>::value) {

354

355  double g1 = 0;

356  double g2 = 0;

357

358  stan::math::gradRegIncBeta(g1, g2, 0.5, 0.5 \* nu\_dbl, r,

359  digammaHalf, digammaNu\_vec[n],

360  digammaNuPlusHalf\_vec[n],

361  betaNuHalf\_vec[n]);

362

363  operands\_and\_partials.d\_x2[n]

364  += zJacobian \* ( - d\_ibeta \* (r / t) \* (r / t) + 0.5 \* g2 ) / Pn;

365  }

366  if (!is\_constant\_struct<T\_loc>::value)

367  operands\_and\_partials.d\_x3[n]

368  += - zJacobian \* d\_ibeta \* J \* sigma\_inv / Pn;

369  if (!is\_constant\_struct<T\_scale>::value)

370  operands\_and\_partials.d\_x4[n]

371  += - zJacobian \* d\_ibeta \* J \* sigma\_inv \* t / Pn;

372  }

373  }

374

375  if (!is\_constant\_struct<T\_y>::value)

376  for(size\_t n = 0; n < stan::length(y); ++n)

377  operands\_and\_partials.d\_x1[n] \*= P;

378  if (!is\_constant\_struct<T\_dof>::value)

379  for(size\_t n = 0; n < stan::length(nu); ++n)

380  operands\_and\_partials.d\_x2[n] \*= P;

381  if (!is\_constant\_struct<T\_loc>::value)

382  for(size\_t n = 0; n < stan::length(mu); ++n)

383  operands\_and\_partials.d\_x3[n] \*= P;

384  if (!is\_constant\_struct<T\_scale>::value)

385  for(size\_t n = 0; n < stan::length(sigma); ++n)

386  operands\_and\_partials.d\_x4[n] \*= P;

387

388  return operands\_and\_partials.to\_var(P);

389  }

390

391  template <typename T\_y, typename T\_dof, typename T\_loc, typename T\_scale>

392  typename return\_type<T\_y, T\_dof, T\_loc, T\_scale>::type

393  student\_t\_cdf\_log(const T\_y& y, const T\_dof& nu, const T\_loc& mu,

394  const T\_scale& sigma) {

395

396  // Size checks

397  if (!(stan::length(y) && stan::length(nu) && stan::length(mu)

398  && stan::length(sigma)))

399  return 0.0;

400

401  static const char\* function = "stan::prob::student\_t\_cdf\_log(%1%)";

402

403  using stan::math::check\_positive\_finite;

404  using stan::math::check\_finite;

405  using stan::math::check\_not\_nan;

406  using stan::math::check\_consistent\_sizes;

407  using stan::math::value\_of;

408

409  double P(0.0);

410

411  check\_not\_nan(function, y, "Random variable", &P);

412  check\_positive\_finite(function, nu, "Degrees of freedom parameter", &P);

413  check\_finite(function, mu, "Location parameter", &P);

414  check\_positive\_finite(function, sigma, "Scale parameter", &P);

415

416  // Wrap arguments in vectors

417  VectorView<const T\_y> y\_vec(y);

418  VectorView<const T\_dof> nu\_vec(nu);

419  VectorView<const T\_loc> mu\_vec(mu);

420  VectorView<const T\_scale> sigma\_vec(sigma);

421  size\_t N = max\_size(y, nu, mu, sigma);

422

423  agrad::OperandsAndPartials<T\_y, T\_dof, T\_loc, T\_scale>

424  operands\_and\_partials(y, nu, mu, sigma);

425

426  // Explicit return for extreme values

427  // The gradients are technically ill-defined, but treated as zero

428  for (size\_t i = 0; i < stan::length(y); i++) {

429  if (value\_of(y\_vec[i]) == -std::numeric\_limits<double>::infinity())

430  return operands\_and\_partials.to\_var(stan::math::negative\_infinity());

431  }

432

433  using boost::math::ibeta;

434  using boost::math::ibeta\_derivative;

435  using boost::math::digamma;

436  using boost::math::beta;

437

438  // Cache a few expensive function calls if nu is a parameter

439  double digammaHalf = 0;

440

441  DoubleVectorView<!is\_constant\_struct<T\_dof>::value,is\_vector<T\_dof>::value>

442  digamma\_vec(stan::length(nu));

443  DoubleVectorView<!is\_constant\_struct<T\_dof>::value,is\_vector<T\_dof>::value>

444  digammaNu\_vec(stan::length(nu));

445  DoubleVectorView<!is\_constant\_struct<T\_dof>::value,is\_vector<T\_dof>::value>

446  digammaNuPlusHalf\_vec(stan::length(nu));

447  DoubleVectorView<!is\_constant\_struct<T\_dof>::value,is\_vector<T\_dof>::value>

448  betaNuHalf\_vec(stan::length(nu));

449

450  if (!is\_constant\_struct<T\_dof>::value) {

451  digammaHalf = digamma(0.5);

452

453  for (size\_t i = 0; i < stan::length(nu); i++) {

454  const double nu\_dbl = value\_of(nu\_vec[i]);

455

456  digammaNu\_vec[i] = digamma(0.5 \* nu\_dbl);

457  digammaNuPlusHalf\_vec[i] = digamma(0.5 + 0.5 \* nu\_dbl);

458  betaNuHalf\_vec[i] = beta(0.5, 0.5 \* nu\_dbl);

459  }

460  }

461

462  // Compute vectorized cdf\_log and gradient

463  for (size\_t n = 0; n < N; n++) {

464

465  // Explicit results for extreme values

466  // The gradients are technically ill-defined, but treated as zero

467  if (value\_of(y\_vec[n]) == std::numeric\_limits<double>::infinity()) {

468  continue;

469  }

470

471  const double sigma\_inv = 1.0 / value\_of(sigma\_vec[n]);

472  const double t = (value\_of(y\_vec[n]) - value\_of(mu\_vec[n])) \* sigma\_inv;

473  const double nu\_dbl = value\_of(nu\_vec[n]);

474  const double q = nu\_dbl / (t \* t);

475  const double r = 1.0 / (1.0 + q);

476  const double J = 2 \* r \* r \* q / t;

477  double zJacobian = t > 0 ? - 0.5 : 0.5;

478

479  if(q < 2) {

480  double z = ibeta(0.5 \* nu\_dbl, 0.5, 1.0 - r);

481  const double Pn = t > 0 ? 1.0 - 0.5 \* z : 0.5 \* z;

482  const double d\_ibeta = ibeta\_derivative(0.5 \* nu\_dbl, 0.5, 1.0 - r);

483

484  P += log(Pn);

485

486  if (!is\_constant\_struct<T\_y>::value)

487  operands\_and\_partials.d\_x1[n]

488  += - zJacobian \* d\_ibeta \* J \* sigma\_inv / Pn;

489

490  if (!is\_constant\_struct<T\_dof>::value) {

491

492  double g1 = 0;

493  double g2 = 0;

494

495  stan::math::gradRegIncBeta(g1, g2, 0.5 \* nu\_dbl, 0.5, 1.0 - r,

496  digammaNu\_vec[n], digammaHalf,

497  digammaNuPlusHalf\_vec[n],

498  betaNuHalf\_vec[n]);

499

500  operands\_and\_partials.d\_x2[n]

501  += zJacobian \* ( d\_ibeta \* (r / t) \* (r / t) + 0.5 \* g1 ) / Pn;

502  }

503

504  if (!is\_constant\_struct<T\_loc>::value)

505  operands\_and\_partials.d\_x3[n]

506  += zJacobian \* d\_ibeta \* J \* sigma\_inv / Pn;

507  if (!is\_constant\_struct<T\_scale>::value)

508  operands\_and\_partials.d\_x4[n]

509  += zJacobian \* d\_ibeta \* J \* sigma\_inv \* t / Pn;

510

511  }

512  else {

513

514  double z = 1 - ibeta(0.5, 0.5 \* nu\_dbl, r);

515  zJacobian \*= -1;

516

517  const double Pn = t > 0 ? 1.0 - 0.5 \* z : 0.5 \* z;

518

519  double d\_ibeta = ibeta\_derivative(0.5, 0.5 \* nu\_dbl, r);

520

521  P += log(Pn);

522

523  if (!is\_constant\_struct<T\_y>::value)

524  operands\_and\_partials.d\_x1[n]

525  += zJacobian \* d\_ibeta \* J \* sigma\_inv / Pn;

526

527  if (!is\_constant\_struct<T\_dof>::value) {

528

529  double g1 = 0;

530  double g2 = 0;

531

532  stan::math::gradRegIncBeta(g1, g2, 0.5, 0.5 \* nu\_dbl, r,

533  digammaHalf, digammaNu\_vec[n],

534  digammaNuPlusHalf\_vec[n],

535  betaNuHalf\_vec[n]);

536

537  operands\_and\_partials.d\_x2[n]

538  += zJacobian \* ( - d\_ibeta \* (r / t) \* (r / t) + 0.5 \* g2 ) / Pn;

539  }

540

541  if (!is\_constant\_struct<T\_loc>::value)

542  operands\_and\_partials.d\_x3[n]

543  += - zJacobian \* d\_ibeta \* J \* sigma\_inv / Pn;

544  if (!is\_constant\_struct<T\_scale>::value)

545  operands\_and\_partials.d\_x4[n]

546  += - zJacobian \* d\_ibeta \* J \* sigma\_inv \* t / Pn;

547  }

548  }

549

550  return operands\_and\_partials.to\_var(P);

551  }

552

553  template <typename T\_y, typename T\_dof, typename T\_loc, typename T\_scale>

554  typename return\_type<T\_y, T\_dof, T\_loc, T\_scale>::type

555  student\_t\_ccdf\_log(const T\_y& y, const T\_dof& nu, const T\_loc& mu,

556  const T\_scale& sigma) {

557

558  // Size checks

559  if (!(stan::length(y) && stan::length(nu) && stan::length(mu)

560  && stan::length(sigma)))

561  return 0.0;

562

563  static const char\* function = "stan::prob::student\_t\_ccdf\_log(%1%)";

564

565  using stan::math::check\_positive\_finite;

566  using stan::math::check\_finite;

567  using stan::math::check\_not\_nan;

568  using stan::math::check\_consistent\_sizes;

569  using stan::math::value\_of;

570

571  double P(0.0);

572

573  check\_not\_nan(function, y, "Random variable", &P);

574  check\_positive\_finite(function, nu, "Degrees of freedom parameter", &P);

575  check\_finite(function, mu, "Location parameter", &P);

576  check\_positive\_finite(function, sigma, "Scale parameter", &P);

577

578  // Wrap arguments in vectors

579  VectorView<const T\_y> y\_vec(y);

580  VectorView<const T\_dof> nu\_vec(nu);

581  VectorView<const T\_loc> mu\_vec(mu);

582  VectorView<const T\_scale> sigma\_vec(sigma);

583  size\_t N = max\_size(y, nu, mu, sigma);

584

585  agrad::OperandsAndPartials<T\_y, T\_dof, T\_loc, T\_scale>

586  operands\_and\_partials(y, nu, mu, sigma);

587

588  // Explicit return for extreme values

589  // The gradients are technically ill-defined, but treated as zero

590  for (size\_t i = 0; i < stan::length(y); i++) {

591  if (value\_of(y\_vec[i]) == -std::numeric\_limits<double>::infinity())

592  return operands\_and\_partials.to\_var(0.0);

593  }

594

595  using boost::math::ibeta;

596  using boost::math::ibeta\_derivative;

597  using boost::math::digamma;

598  using boost::math::beta;

599

600  // Cache a few expensive function calls if nu is a parameter

601  double digammaHalf = 0;

602

603  DoubleVectorView<!is\_constant\_struct<T\_dof>::value,is\_vector<T\_dof>::value>

604  digamma\_vec(stan::length(nu));

605  DoubleVectorView<!is\_constant\_struct<T\_dof>::value,is\_vector<T\_dof>::value>

606  digammaNu\_vec(stan::length(nu));

607  DoubleVectorView<!is\_constant\_struct<T\_dof>::value,is\_vector<T\_dof>::value>

608  digammaNuPlusHalf\_vec(stan::length(nu));

609  DoubleVectorView<!is\_constant\_struct<T\_dof>::value,is\_vector<T\_dof>::value>

610  betaNuHalf\_vec(stan::length(nu));

611

612  if (!is\_constant\_struct<T\_dof>::value) {

613  digammaHalf = digamma(0.5);

614

615  for (size\_t i = 0; i < stan::length(nu); i++) {

616  const double nu\_dbl = value\_of(nu\_vec[i]);

617

618  digammaNu\_vec[i] = digamma(0.5 \* nu\_dbl);

619  digammaNuPlusHalf\_vec[i] = digamma(0.5 + 0.5 \* nu\_dbl);

620  betaNuHalf\_vec[i] = beta(0.5, 0.5 \* nu\_dbl);

621  }

622  }

623

624  // Compute vectorized cdf\_log and gradient

625  for (size\_t n = 0; n < N; n++) {

626

627  // Explicit results for extreme values

628  // The gradients are technically ill-defined, but treated as zero

629  if (value\_of(y\_vec[n]) == std::numeric\_limits<double>::infinity()) {

630  return operands\_and\_partials.to\_var(stan::math::negative\_infinity());

631  }

632

633  const double sigma\_inv = 1.0 / value\_of(sigma\_vec[n]);

634  const double t = (value\_of(y\_vec[n]) - value\_of(mu\_vec[n])) \* sigma\_inv;

635  const double nu\_dbl = value\_of(nu\_vec[n]);

636  const double q = nu\_dbl / (t \* t);

637  const double r = 1.0 / (1.0 + q);

638  const double J = 2 \* r \* r \* q / t;

639  double zJacobian = t > 0 ? - 0.5 : 0.5;

640

641  if(q < 2) {

642  double z = ibeta(0.5 \* nu\_dbl, 0.5, 1.0 - r);

643  const double Pn = t > 0 ? 0.5 \* z : 1.0 - 0.5 \* z;

644  const double d\_ibeta = ibeta\_derivative(0.5 \* nu\_dbl, 0.5, 1.0 - r);

645

646  P += log(Pn);

647

648  if (!is\_constant\_struct<T\_y>::value)

649  operands\_and\_partials.d\_x1[n]

650  += zJacobian \* d\_ibeta \* J \* sigma\_inv / Pn;

651

652  if (!is\_constant\_struct<T\_dof>::value) {

653

654  double g1 = 0;

655  double g2 = 0;

656

657  stan::math::gradRegIncBeta(g1, g2, 0.5 \* nu\_dbl, 0.5, 1.0 - r,

658  digammaNu\_vec[n], digammaHalf,

659  digammaNuPlusHalf\_vec[n],

660  betaNuHalf\_vec[n]);

661

662  operands\_and\_partials.d\_x2[n]

663  -= zJacobian \* ( d\_ibeta \* (r / t) \* (r / t) + 0.5 \* g1 ) / Pn;

664  }

665

666  if (!is\_constant\_struct<T\_loc>::value)

667  operands\_and\_partials.d\_x3[n]

668  -= zJacobian \* d\_ibeta \* J \* sigma\_inv / Pn;

669  if (!is\_constant\_struct<T\_scale>::value)

670  operands\_and\_partials.d\_x4[n]

671  -= zJacobian \* d\_ibeta \* J \* sigma\_inv \* t / Pn;

672

673  }

674  else {

675

676  double z = 1 - ibeta(0.5, 0.5 \* nu\_dbl, r);

677  zJacobian \*= -1;

678

679  const double Pn = t > 0 ? 0.5 \* z : 1.0 - 0.5 \* z;

680

681  double d\_ibeta = ibeta\_derivative(0.5, 0.5 \* nu\_dbl, r);

682

683  P += log(Pn);

684

685  if (!is\_constant\_struct<T\_y>::value)

686  operands\_and\_partials.d\_x1[n]

687  -= zJacobian \* d\_ibeta \* J \* sigma\_inv / Pn;

688

689  if (!is\_constant\_struct<T\_dof>::value) {

690

691  double g1 = 0;

692  double g2 = 0;

693

694  stan::math::gradRegIncBeta(g1, g2, 0.5, 0.5 \* nu\_dbl, r,

695  digammaHalf, digammaNu\_vec[n],

696  digammaNuPlusHalf\_vec[n],

697  betaNuHalf\_vec[n]);

698

699  operands\_and\_partials.d\_x2[n]

700  -= zJacobian \* ( - d\_ibeta \* (r / t) \* (r / t) + 0.5 \* g2 ) / Pn;

701  }

702

703  if (!is\_constant\_struct<T\_loc>::value)

704  operands\_and\_partials.d\_x3[n]

705  += zJacobian \* d\_ibeta \* J \* sigma\_inv / Pn;

706  if (!is\_constant\_struct<T\_scale>::value)

707  operands\_and\_partials.d\_x4[n]

708  += zJacobian \* d\_ibeta \* J \* sigma\_inv \* t / Pn;

709  }

710  }

711

712  return operands\_and\_partials.to\_var(P);

713  }

714

715  template <class RNG>

716  inline double

717  student\_t\_rng(const double nu,

718  const double mu,

719  const double sigma,

720  RNG& rng) {

721  using boost::variate\_generator;

722  using boost::random::student\_t\_distribution;

723

724  static const char\* function = "stan::prob::student\_t\_rng(%1%)";

725

726  using stan::math::check\_positive\_finite;

727  using stan::math::check\_finite;

728

729  check\_positive\_finite(function, nu, "Degrees of freedom parameter",

730  (double\*)0);

731  check\_finite(function, mu, "Location parameter", (double\*)0);

732  check\_positive\_finite(function, sigma, "Scale parameter", (double\*)0);

733

734  variate\_generator<RNG&, student\_t\_distribution<> >

735  rng\_unit\_student\_t(rng, student\_t\_distribution<>(nu));

736  return mu + sigma \* rng\_unit\_student\_t();

737  }

738  }

739 }

740 #endif

stan::math::square

T square(const T x)

Return the square of the specified argument.

**Definition:** square.hpp:22

stan::agrad::log1p\_exp

fvar< T > log1p\_exp(const fvar< T > &x)

**Definition:** log1p\_exp.hpp:15

stan::math::gradRegIncBeta

void gradRegIncBeta(double &g1, double &g2, double a, double b, double z, double digammaA, double digammaB, double digammaSum, double betaAB)

**Definition:** internal\_math.hpp:161

traits.hpp

stan::prob::student\_t\_cdf

return\_type< T\_y, T\_dof, T\_loc, T\_scale >::type student\_t\_cdf(const T\_y &y, const T\_dof &nu, const T\_loc &mu, const T\_scale &sigma)

**Definition:** student\_t.hpp:219

stan::agrad::OperandsAndPartials::to\_var

T\_return\_type to\_var(double logp)

**Definition:** partials\_vari.hpp:138

stan::math::check\_positive\_finite

bool check\_positive\_finite(const char \*function, const T\_y &y, const char \*name, T\_result \*result)

**Definition:** check\_positive\_finite.hpp:12

error\_handling.hpp

square.hpp

stan::length

size\_t length(const T &)

**Definition:** traits.hpp:159

stan::agrad::square

fvar< T > square(const fvar< T > &x)

**Definition:** square.hpp:15

stan::DoubleVectorView

DoubleVectorView allocates double values to be used as intermediate values.

**Definition:** traits.hpp:358

stan::math::check\_finite

bool check\_finite(const char \*function, const T\_y &y, const char \*name, T\_result \*result)

Checks if the variable y is finite.

**Definition:** check\_finite.hpp:52

partials\_vari.hpp

stan::agrad::value\_of

T value\_of(const fvar< T > &v)

Return the value of the specified variable.

**Definition:** value\_of.hpp:16

constants.hpp

stan::is\_vector

**Definition:** traits.hpp:83

stan::agrad::lgamma

fvar< T > lgamma(const fvar< T > &x)

**Definition:** lgamma.hpp:15

stan::agrad::OperandsAndPartials

A variable implementation that stores operands and derivatives with respect to the variable...

**Definition:** partials\_vari.hpp:76

traits.hpp

stan::return\_type::type

boost::math::tools::promote\_args< typename scalar\_type< T1 >::type, typename scalar\_type< T2 >::type, typename scalar\_type< T3 >::type, typename scalar\_type< T4 >::type, typename scalar\_type< T5 >::type, typename scalar\_type< T6 >::type >::type type

**Definition:** traits.hpp:406

stan::is\_constant\_struct

Metaprogram to determine if a type has a base scalar type that can be assigned to type double...

**Definition:** traits.hpp:57

stan::math::value\_of

double value\_of(const T x)

Return the value of the specified scalar argument converted to a double value.

**Definition:** value\_of.hpp:24

stan::prob::include\_summand

Template metaprogram to calculate whether a summand needs to be included in a proportional (log) prob...

**Definition:** traits.hpp:35

stan::agrad::OperandsAndPartials::d\_x2

VectorView< double \*, is\_vector< T2 >::value, is\_constant\_struct< T2 >::value > d\_x2

**Definition:** partials\_vari.hpp:83

stan::agrad::ibeta

var ibeta(const var &a, const var &b, const var &x)

The normalized incomplete beta function of a, b, and x.

**Definition:** ibeta.hpp:223

stan::prob::student\_t\_log

return\_type< T\_y, T\_dof, T\_loc, T\_scale >::type student\_t\_log(const T\_y &y, const T\_dof &nu, const T\_loc &mu, const T\_scale &sigma)

The log of the Student-t density for the given y, nu, mean, and scale parameter.

**Definition:** student\_t.hpp:49

stan::math::check\_consistent\_sizes

bool check\_consistent\_sizes(const char \*function, const T1 &x1, const T2 &x2, const char \*name1, const char \*name2, T\_result \*result)

**Definition:** check\_consistent\_sizes.hpp:13

stan::max\_size

size\_t max\_size(const T1 &x1, const T2 &x2)

**Definition:** traits.hpp:191

internal\_math.hpp

stan::math::check\_not\_nan

bool check\_not\_nan(const char \*function, const T\_y &y, const char \*name, T\_result \*result)

Checks if the variable y is nan.

**Definition:** check\_not\_nan.hpp:56

stan::prob::student\_t\_cdf\_log

return\_type< T\_y, T\_dof, T\_loc, T\_scale >::type student\_t\_cdf\_log(const T\_y &y, const T\_dof &nu, const T\_loc &mu, const T\_scale &sigma)

**Definition:** student\_t.hpp:393

stan::agrad::OperandsAndPartials::d\_x4

VectorView< double \*, is\_vector< T4 >::value, is\_constant\_struct< T4 >::value > d\_x4

**Definition:** partials\_vari.hpp:85

stan::agrad::digamma

fvar< T > digamma(const fvar< T > &x)

**Definition:** digamma.hpp:16

value\_of.hpp

stan::agrad::OperandsAndPartials::d\_x1

VectorView< double \*, is\_vector< T1 >::value, is\_constant\_struct< T1 >::value > d\_x1

**Definition:** partials\_vari.hpp:82

stan::agrad::OperandsAndPartials::d\_x3

VectorView< double \*, is\_vector< T3 >::value, is\_constant\_struct< T3 >::value > d\_x3

**Definition:** partials\_vari.hpp:84

stan::prob::student\_t\_ccdf\_log

return\_type< T\_y, T\_dof, T\_loc, T\_scale >::type student\_t\_ccdf\_log(const T\_y &y, const T\_dof &nu, const T\_loc &mu, const T\_scale &sigma)

**Definition:** student\_t.hpp:555

stan::prob::student\_t\_rng

double student\_t\_rng(const double nu, const double mu, const double sigma, RNG &rng)

**Definition:** student\_t.hpp:717

constants.hpp

stan::agrad::log

fvar< T > log(const fvar< T > &x)

**Definition:** log.hpp:15

stan::VectorView

VectorView is a template metaprogram that takes its argument and allows it to be used like a vector...

**Definition:** traits.hpp:275

stan::agrad::log1p

fvar< T > log1p(const fvar< T > &x)

**Definition:** log1p.hpp:16

stan::math::negative\_infinity

double negative\_infinity()

Return negative infinity.

**Definition:** constants.hpp:123


---

[ Stan Home Page ]

*© 2011–2014,
Stan Development Team.*
